# Estimated HPV-Attributable Inpatient Pharmaceutical Reimbursement in Bulgaria, 2021–2025: A National Payer-Perspective Expenditure Analysis

**DOI:** 10.3390/healthcare14162474

**Published:** 2026-08-10

**Authors:** Kostadin Kostadinov, Victoria Mandova, Vanya Rangelova, Ani Kevorkyan, Ralitsa Raycheva

**Affiliations:** 1 Department of Social Medicine and Public Health, Faculty of Public Health, Medical University of Plovdiv, 4002 Plovdiv, Bulgaria; viktoriya.mandova@mu-plovdiv.bg (V.M.); ralitsa.raycheva@mu-plovdiv.bg (R.R.); 2 Environmental Health Division, Research Institute at Medical University of Plovdiv, 15-A “Vasil Aprilov” Blvd., 4002 Plovdiv, Bulgaria; 3 Department of Epidemiology and Disaster Medicine, Faculty of Public Health, Medical University of Plovdiv, 4002 Plovdiv, Bulgaria; vanya.rangelova@mu-plovdiv.bg (V.R.); ani.kevorkyan@mu-plovdiv.bg (A.K.)

**Keywords:** human papillomavirus, cervical cancer, cost of illness, health expenditure, pembrolizumab, Bulgaria, reimbursement, price index decomposition

## Abstract

**Highlights:**

**What are the main findings?**

- Estimated HPV-attributable inpatient antineoplastic reimbursement in Bulgaria rose 5.4-fold in nominal terms, and 4.2-fold in real terms, to nearly EUR 20 million by 2025, with cervical cancer accounting for 81.8%.
- A price–volume–entry decomposition attributed the growth almost entirely to the reimbursement entry of one immunotherapy, pembrolizumab, and not to higher prices or wider use of established drugs.

**What are the implications of the main findings?**

- Because the rise reflects a single high-cost product entering reimbursement, managed-entry agreements and value-based procurement are the proportionate tools for containing it.
- The quantified expenditure provides a cost-of-illness parameter that strengthens the economic case for HPV vaccination and organised cervical screening in a low-coverage setting.

**Abstract:**

**Background**: Human papillomavirus (HPV)-attributable cancers remain largely preventable but continue to generate increasing treatment costs where vaccination and screening coverage are limited. We estimated direct inpatient pharmaceutical reimbursement attributable to HPV-related cancers in Bulgaria and examined the drivers of expenditure growth. **Methods**: National Health Insurance Fund reimbursement data (2021–2025) were analysed for antineoplastic medicines used to treat eight HPV-associated cancer sites. Site-specific population-attributable fractions (PAFs) were applied to estimate HPV-attributable expenditure. Uncertainty in PAFs was quantified using 10,000 Monte Carlo simulations and discrete attribution scenarios. Expenditure growth was decomposed into price, volume, and net product-entry/exit effects using Fisher indices. Expenditure was additionally expressed in constant 2021 euros using the Harmonised Index of Consumer Prices. **Results**: Estimated HPV-attributable inpatient pharmaceutical reimbursement totalled EUR 54.2 million (probabilistic median; 95% uncertainty interval: EUR 53.3–55.0 million). Cervical cancer accounted for 81.8% of the total. Results varied by less than ±6% across attribution scenarios and remained EUR 44.6 million when head-and-neck cancers were excluded. Annual expenditure increased from EUR 3.67 million in 2021 to EUR 19.89 million in 2025, representing a 5.4-fold nominal and 4.2-fold inflation-adjusted increase. Growth was concentrated in cervical cancer and was driven primarily by the introduction of new therapies rather than higher prices or greater use of existing medicines. Net product entry explained 88.8% of total log growth, while reimbursement prices for continuing products declined by approximately 31%. Pembrolizumab, first reimbursed for cervical cancer in 2023, accounted for EUR 16.03 million of cervical cancer expenditure in 2025. **Conclusions**: These ecological estimates indicate a rapid increase in HPV-attributable inpatient pharmaceutical reimbursement in Bulgaria, largely driven by cervical cancer and the introduction of immunotherapy. The findings have implications for budget forecasting, procurement planning, and economic evaluation of HPV prevention strategies.

## 1. Introduction

Persistent infection with oncogenic human papillomavirus (HPV) genotypes is the necessary cause of cervical cancer and a major contributor to cancers of the vulva, vagina, anus, penis, and oropharynx [1,2]. HPV accounts for several hundred thousand new cancers worldwide each year, and the attributable fraction varies markedly by anatomical site, ranging from effectively complete attribution for the cervix to a minority of laryngeal and oral-cavity tumours [1,2,3]. The most recent global estimate attributes approximately 831,000 cancer cases and 423,000 deaths to HPV in 2022, of which cervical cancer constitutes about three quarters [3]. Because the great majority of this burden is in principle preventable through prophylactic vaccination and organised cervical screening, the persistence of HPV-attributable malignancy in a given health system can be read as a marker of incompletely realised prevention, and the expenditure it generates represents a partly avoidable claim on finite public resources [1,4,5].

The contrast between settings that have achieved high vaccination coverage and those that have not is increasingly stark. Population-level data from England, Scotland, the Netherlands, and Australia document substantial declines in cervical intraepithelial neoplasia and invasive cervical cancer in vaccinated birth cohorts, together with herd effects in unvaccinated individuals [4,6]. Bulgaria occupies the opposite position within the European Union. It combines one of the highest cervical cancer incidence and mortality rates in the bloc with persistently low HPV vaccination uptake, full-course coverage having remained well below 15% of the eligible cohort, and with the absence of an organised, population-based screening programme [7,8]. The country therefore carries a disease burden that other European systems are actively retiring, and it does so while its treatment armamentarium is being transformed by the arrival of high-cost biologic and immunotherapeutic agents. The pharmaceutical reimbursement expenditure that this persisting burden generates has not, however, been quantified from complete national data, and the components of any growth in it have not been separated. Establishing that expenditure, and resolving its growth into interpretable price, volume, and product-entry parts, is the gap the present analysis addresses.

The economic dimension of HPV-related cancer is well characterised internationally, though unevenly across cost components and sites. National cost-of-illness studies converge on a few regularities. Costs concentrate in the first year after diagnosis [9,10]. Cervical cancer dominates the female burden, while oropharyngeal and other head-and-neck cancers account for a growing and largely male-driven share of European spending [11,12,13,14]. Indirect productivity losses frequently exceed direct medical costs [11,15,16]. Reported national totals span a wide range, from tens of millions of euros per year for treatment in smaller high-income systems to several hundred million across broader condition sets and some EUR 9 billion for all HPV-associated disease in the United States [10,11,13,17,18,19]. That spread reflects differences in scope and method rather than in underlying epidemiology. For Central and Eastern Europe, the future productivity loss from HPV-related cancer deaths has been estimated at EUR 4–46 million per country, around EUR 7 million for Bulgaria from a restricted set of sites [15].

A recurrent and policy-relevant finding in this literature concerns the changing composition of cost over time. In the United States, total HPV-related direct medical cost has remained near EUR 9 billion despite vaccination-era declines in some cancers, because rising per-case treatment cost has offset falling incidence. In the most recent national accounting, treatment of HPV-attributable cancer rose to represent 43.8% of the total, against 55.0% for routine screening and follow-up [18]. This shift, from prevention-dominated to treatment-dominated spending driven by newer and more expensive therapies, is the macro-level signal that the present analysis examines at the level of an individual middle-income payer.

For Bulgaria, prior national estimates addressed the total economic burden of cervical cancer including productivity losses [7] and the non-cervical HPV-associated sites in a companion analysis [20], but neither isolated the pharmaceutical component from complete reimbursement data nor decomposed its growth into more reimbursement claims, higher prices for established therapies, or the entry of new and expensive molecules. The distinction matters beyond Bulgaria. Oncology drug expenditure is rising across health systems, and the immunotherapy escalation documented in high-income settings is increasingly reaching middle-income payers [21], yet administrative reimbursement studies rarely separate that growth into interpretable price, volume, and entry components, and the few that do have generally attributed it to expanding volume rather than to new-product entry [22,23]. A transparent decomposition applied to complete national data attributes budgetary change to its proximate source. That attribution is central to forecasting and procurement, and it yields the cost-of-illness parameter that any later cost-effectiveness evaluation of gender-neutral HPV vaccination in Bulgaria will require. No prior Bulgarian study has provided it.

We therefore analysed five complete calendar years of inpatient antineoplastic reimbursement records from the Bulgarian National Health Insurance Fund (NHIF). Our objectives were threefold. The first was to estimate the direct HPV-attributable inpatient pharmaceutical reimbursement expenditure across eight eligible cancer sites with propagated uncertainty. The second was to characterise its temporal trajectory and locate any change in slope. The third was to decompose the observed growth into interpretable price, volume, and product-entry components, so that the components of the change, and not only its magnitude, are made explicit.

## 2. Materials and Methods

### 2.1. Study Design and Reporting

This was a retrospective, longitudinal, payer-perspective analysis of routinely collected administrative reimbursement data, conducted from the perspective of the public payer. It is an ecological attribution exercise rather than a cross-sectional epidemiological study, because human papillomavirus status is not ascertained in the individual record and attribution is made at the level of the cancer site rather than the tumour (Section 2.3). Three economic quantities are kept distinct and named consistently throughout. Reimbursement is the amount the National Health Insurance Fund paid for a dispensed product. Expenditure is the aggregate of that reimbursement across strata, sites, or years. The unqualified word cost is reserved for the broader economic concepts (total direct medical cost, per-case cost, and the cost-of-illness parameter) that this analysis does not measure. The work is described as an expenditure analysis rather than a cost-of-illness study advisedly, because the data capture reimbursed inpatient antineoplastic pharmaceutical expenditure under one financing instrument rather than the societal or full direct medical cost of disease, and because the unit of observation is a monthly reimbursement stratum rather than an incident or prevalent case. Reporting follows the Consolidated Health Economic Evaluation Reporting Standards (CHEERS 2022) for the economic component [24] and, because the data are collected routinely for administration rather than for research, the REporting of studies Conducted using Observational Routinely collected health Data (RECORD) statement, the routinely collected-data extension of the STROBE statement, for the observational component [25,26]. The unit of observation was the administrative stratum defined by the combination of regional health insurance office, contracted hospital, antineoplastic product (NHIF product code), ICD-10 diagnosis, and calendar month. The completed CHEERS and RECORD checklists are provided as Supplementary S10 and S11.

### 2.2. Data Source

The data source was the NHIF inpatient pharmaceutical reimbursement database, known administratively as Application 2 (outside the value of the clinical pathway; hereafter NHIF Application 2). It records antineoplastic and supportive medicines dispensed to hospitalised patients outside the bundled clinical-pathway tariff, and the dataset is publicly available via Zenodo [27]. Eligibility was defined by diagnosis rather than by drug class. Every product billed under an eligible ICD-10 code (Section 2.3) was retained, whether antineoplastic or supportive, so that the reimbursement attributable to a cancer site is captured in full rather than restricted to a pre-selected list of agents. The released file covers July 2020 through December 2025. The analysis was restricted to the five complete calendar years 2021–2025 (60 months), excluding the partial July–December 2020 window because of its asymmetry and the documented early-pandemic suppression of cancer treatment initiation across European systems.

### 2.3. Cancer Site Scope and Population-Attributable Fractions

Eligible HPV-attributable cancer sites were defined by ICD-10 code and assigned base-case population-attributable fractions (PAFs) drawn from the pooled international literature [1,2,10,11,12] (Supplementary S2). The cervical PAF was fixed at 1.00 and not varied, consistent with HPV being the necessary cause of cervical carcinoma. Records were selected by applying the case-insensitive, whitespace-trimmed regular expression ^(C53|C51|C52|C21|C60|C01|C09|C10) to standardised ICD-10 codes. Larynx (C32) and oral cavity (C02–C06), which carry a substantially lower and more heterogeneous HPV-attributable fraction, were excluded from the base case. The head-and-neck attribution scenarios (Supplementary S5) bound the influence of the included head-and-neck sites on the aggregate. Because the database covers invasive malignancies receiving antineoplastic therapy, carcinoma-in situ codes do not appear and required no explicit exclusion.

The attribution strategy is ecological rather than tumour-level. HPV status is not ascertained in the reimbursement record, so site-level fractions are applied to aggregate expenditure to obtain an attributable estimate, expenditure × PAF. This is a standard and pragmatic device in HPV cost-of-illness work [10,11,12], but it carries two distinct kinds of uncertainty. The first is parameter uncertainty in the fractions themselves, which we propagate probabilistically (Section 2.9). The second is structural. The true attributable fraction varies geographically, temporally, and by sex, smoking prevalence, and histology, most consequentially at the oropharyngeal, tonsillar, and base-of-tongue sites, where reported HPV positivity is heterogeneous across European populations. The PAFs applied here are derived from pooled international estimates and have not been validated against Bulgarian or South-Eastern European genotyping data. Given Bulgaria’s historically low vaccination coverage and the influence of population-specific cofactors on the attributable fraction at non-cervical sites, their transportability is uncertain. We therefore do not treat the probabilistic interval as a complete characterisation of attribution uncertainty, and we additionally examine discrete attribution scenarios (Section 2.9) to establish whether the substantive conclusions survive deliberate variation of the assumption rather than only sampling around a fixed point. The cervical fraction, fixed at unity throughout because HPV is the necessary cause of cervical carcinoma, is the one component not subject to this concern, and it dominates the aggregate.

### 2.4. Data Preparation

Data preparation proceeded in a fixed, auditable sequence, with record counts reported at each step (Supplementary S1). After ingestion and restriction to 2021–2025, the raw file comprised 943,169 billing lines across 54 contracted hospitals, 16 administering regional health insurance offices, and 487 distinct ICD-10 codes, totalling EUR 2,910,470,761 in observed expenditure across all diagnoses. The product-code, quantity, and expenditure fields were complete, with no missing values, and the reimbursed amounts contained no negative entries, corrections, or reversals. A missing ICD-10 diagnosis was present in 6698 lines. These cannot match the site regular expression (Section 2.3) and therefore fall outside the HPV scope by construction, remaining only in the all-diagnoses total used for the denominator. Zero-cost records (n = 588, 0.06%) were excluded. Twenty-four of these lay within the HPV scope and six carried a non-zero pack quantity (six packs in total, 0.004% of HPV-scope pack volume), so their removal is immaterial to the volume decomposition and additionally protects the per-pack price identification, which a zero-priced pack would corrupt. A cost-outlier review flagged records exceeding ten times the 99th percentile of stratum-level expenditure (threshold EUR 477,284). The ten flagged records were clinically plausible high-cost dispensings (eptacog alfa for congenital factor VIII deficiency and enzalutamide for prostate cancer), lay outside the HPV scope, and were retained. The same rule was applied within the HPV scope, where no stratum reached the threshold. The largest HPV-scope stratum was a single-month pembrolizumab cervical administration of EUR 178,528 (62 packs, EUR 2879 per pack). Pembrolizumab per-pack reimbursement was tightly clustered (interquartile range approximately EUR 2867 to EUR 2893, full range EUR 2804 to EUR 3068; Supplementary S1) and consistent with the reimbursed unit price, so no data-entry outlier inflates the product-entry effect that drives the cervical result. ICD-10 and Anatomical Therapeutic Chemical (ATC) codes were standardised by uppercasing and whitespace trimming, and Cyrillic or Latin homoglyphs were repaired. A total of 140 NHIF product codes carried more than one ATC label, reflecting the legacy-versus-current WHO ATC reclassification of several monoclonal antibodies and protein-kinase inhibitors (for example, bevacizumab L01XC07 to L01FG01 and pembrolizumab L01XC18 to L01FF02). Each was canonicalised to a single modal ATC code and name, yielding 190 distinct ATC codes (Supplementary S8). Finally, billing lines were collapsed to the documented facility–product–diagnosis–month stratum by summing the additive payment attributes. The 2267 multi-line strata (at most two lines each) were summed rather than deleted, a reconciliation verified to be loss-free for total expenditure to the cent and producing 940,314 unique strata. This collapse is a reconciliation to the publisher’s analytical unit and not an exclusion. The full attrition table and data-handling detail are given in Supplementary S1.

### 2.5. Outcome Derivation

The primary outcome was HPV-attributable antineoplastic pharmaceutical expenditure in euros. For each stratum, HPV-attributable expenditure was computed as observed expenditure multiplied by the site-specific base-case PAF, with low and high bounds obtained by substituting the low and high PAFs. HPV-attributable administration counts were derived analogously. All expenditure figures are nominal euros unless explicitly described as constant-price (Section 2.7).

### 2.6. Population Denominators

National Statistical Institute mid-year resident population estimates were mapped from the 28 administrative districts to the 16 NHIF regional offices represented in the data using a documented crosswalk that assigns each secondary district to its parent office. The Pernik regional office bills under no code in the released extract, so its resident population was excluded from the per capita denominators in order that numerator and denominator range over the same 16 represented offices. The resulting national denominator supports the per capita expenditure series (Section 2.7). Because the regional field encodes the administering office of the treating hospital rather than patient residence, any population-scaled figure is a service-utilisation density and not an incidence rate.

### 2.7. Real-Terms and per Capita Adjustment

To distinguish genuine growth in reimbursement from artefacts of general price inflation and of population change, two further adjustments were applied to the all-site annual series. First, the nominal series was re-expressed in constant 2021 euros using the Bulgarian all-item Harmonised Index of Consumer Prices (HICP, Eurostat series prc_hicp_aind, CP0000BG), rebased to 2021 = 100 [28]. This is conceptually distinct from the within-product Fisher price index of Section 2.8. The Fisher index removes movement in administratively set reimbursement prices, which in fact fell over the period, whereas the HICP removes economy-wide consumer-price inflation, so that a real series which still rises steeply after HICP deflation cannot be a general price-level artefact. Second, a denominator-based national series expressed annual HPV-attributable expenditure per 100,000 of the represented resident population, so that growth in the rate of system expenditure could be separated from any growth attributable to population change. This per capita series is a service-utilisation density computed on the administering-office population and is not an incidence rate. Both series are tabulated in Supplementary S4.

### 2.8. Price–Volume–Entry Decomposition

Observed expenditure is the product of reimbursement price and quantity, and the dataset records the reimbursed pack quantity jointly with the amount paid, so price is identified internally at the product level. The change in expenditure between a base and a comparison year was decomposed into three multiplicatively exact components: a within-product price effect, a within-product volume effect, and a net product-entry or exit effect. This separation matters for policy, because price inflation in existing products, wider use of established regimens, and the entry of new high-cost molecules call for different instruments (price renegotiation, volume management, and managed-entry agreements respectively), so attributing growth to the correct component is a precondition for a proportionate response. Volume is defined precisely as the reimbursed pack quantity summed within an NHIF product code. It is a count of reimbursed packs and not of patients, administrations, claims, or standard units, so stable volume means stable pack throughput of established products and must not be read as evidence about patient demand. The within-product price and volume effects were measured with Fisher indices (the geometric mean of the Laspeyres and Paasche indices), and cross-checked against the Törnqvist index [29], while the entry effect captured molecules present in only one of the two years. Because Fisher indices satisfy the factor-reversal test, the logarithmic identity ln(V1/V0) = ln PF + ln QF + ln Rentry holds exactly, and the numerical validation against synthetic panels with known invariants is reported in Supplementary S6. The decomposition unit was the NHIF product code, within which the pack is homogeneous, so expenditure per pack is a genuine per-pack price free of unit-value composition bias. Because the PAF is a time-invariant constant within a site, it cancels in every index ratio, so the decomposition of observed expenditure is identical to that of HPV-attributable expenditure and, for the cervix (PAF = 1.00), the two coincide exactly. Distinct product codes for one molecule, including reference biologics and their biosimilars, are legitimately separate products, so biosimilar entry registers in the entry component rather than as a within-product price decline. No external price deflator was applied to the decomposition. The constant-price (real) cervical expenditure series was derived internally from the Fisher price index, while the separate economy-wide HICP deflation is described in Section 2.7.

### 2.9. Statistical Analysis

Monthly HPV-attributable expenditure is continuous, strictly positive, right-skewed, and serially autocorrelated. Its monthly trajectory was summarised descriptively by a generalised least squares regression of log expenditure on a calendar-month index with a first-order autoregressive error structure, treated as a smoothing device rather than as an inferential model. This choice was deliberate. Across the 60 months the log-series behaves almost as a random walk, and its residual autocorrelation is not fully cleared by the autoregressive correction, so the effective number of independent observations is small and any confidence interval or *p*-value on the monthly slope would understate the true uncertainty. A higher-order autoregressive or ARMA specification was not pursued, because the residual structure originates in the trend and the mid-series regime change rather than in the autoregressive order, and would not be expected to yield a valid interval on 60 points spanning a structural break. The monthly cost ratio (the exponentiated month coefficient) is therefore reported as a descriptive average increment without an inferential interval, and the autoregressive parameter, the lag-1 residual autocorrelation, and the Ljung–Box statistic are reported alongside it so that its limitations remain auditable (Supplementary S9). Gamma, quasi-Poisson, log-linear ordinary least squares, and negative-binomial fits are retained only as independent-error comparators and read as an upper bound on the increment. The substantive temporal evidence rests on the descriptive annual series and the growth decomposition, not on any fitted monthly slope, and the monthly ratio is never interpreted as an incidence rate ratio.

A change in the slope of monthly cervical expenditure was characterised with segmented (piecewise linear) regression estimating a single breakpoint [30]. We treat this as an exploratory, descriptive localisation rather than as a confirmatory test of a structural break. A single breakpoint was specified a priori because the candidate perturbation (entry of a new molecule) is singular, no multiple-breakpoint search was conducted, and the breakpoint confidence interval is conditional on independent errors and therefore likely anticonservative. A log-scale fit was run as a robustness check on the location. This caveat is carried through to the Results and Discussion. We did not fit a formal interrupted time series, because the relevant perturbations (pandemic disruption and gradual market entry of new molecules) were diffuse rather than single, sharp, externally imposed interventions [31].

Uncertainty in the PAFs was propagated through a probabilistic sensitivity analysis with 10,000 Monte Carlo draws. For each non-cervical site, the PAF was sampled from a Beta distribution moment-matched to the published point estimate, with the low and high bounds treated as an approximate 95% interval, while the cervical PAF was fixed at unity. The full draw vector of aggregate HPV-attributable cost was persisted for downstream cost-effectiveness modelling, consistent with CHEERS input-parameter requirements [24]. The draw distribution is summarised in Supplementary S3.

Because the probabilistic analysis samples around fixed published values and therefore addresses parameter rather than structural uncertainty, we additionally evaluated four discrete attribution scenarios that vary the assumption itself. A conservative scenario applied the published lower PAF bounds to all non-cervical sites and an optimistic scenario applied the upper bounds. An alternative-literature scenario substituted lower head-and-neck fractions (oropharynx and base of tongue 0.45, tonsil 0.55) to reflect the documented geographic heterogeneity in HPV positivity at these sites. A fourth scenario excluded the three head-and-neck sites entirely (PAF set to zero), isolating the cervical and anogenital burden least subject to transportability concern. The cervical PAF was fixed at unity in every scenario. For each scenario we report the aggregate HPV-attributable expenditure, its percentage deviation from the base case, and the cervical share, so that the robustness of both the magnitude and the compositional dominance of cervical cancer can be judged against deliberate variation of the attribution assumption (Supplementary S5).

Analyses were performed in R version 4.5.0 (R Foundation for Statistical Computing, Vienna, Austria) using the tidyverse, segmented, nlme, and broom packages, together with the in-house kkstatfun package for figure styling. The full analysis code and a pinned environment lockfile are deposited alongside the dataset, and the session record is provided as a Supplementary File.

### 2.10. Ethics and Data Availability

The analysis used a publicly available, fully de-identified administrative dataset aggregated at the facility–product–diagnosis–month level and containing no individual-level identifiers; it therefore did not constitute human-subjects research requiring ethical approval. The dataset is available via Zenodo (DOI: 10.5281/zenodo.19160637) [27], population denominators from the National Statistical Institute open-data portal, and the analysis code from the associated repository.

## 3. Results

### 3.1. Aggregate Burden and Composition

Across the 60-month study period, 40,179 HPV-scope strata (4.27% of all cleaned strata) were identified, spanning 44 hospitals and all 16 represented regional offices and comprising 62,066 unadjusted reimbursement administrations. The estimated total HPV-attributable inpatient pharmaceutical expenditure was EUR 54,150,383, with a one-way PAF interval of EUR 52,807,101 to EUR 55,493,666 (Table 1). The probabilistic sensitivity analysis returned a closely concordant median of EUR 54,161,894 (95% interval EUR 53,292,429 to EUR 54,950,063), an interval that reflects sampling around the published fractions rather than the wider structural uncertainty of the attribution (Section 4.5). Across all diagnoses, the estimated HPV-attributable expenditure represented 1.86% of total NHIF Application 2 expenditure.

Cervical cancer dominated the burden, accounting for EUR 44,288,937, or 81.8% of HPV-attributable expenditure and 80.7% of HPV-attributable administrations. The three head-and-neck sites (oropharynx, tonsil, and base of tongue) together accounted for a further 17.7%, while the four remaining anogenital sites (anus, vulva, vagina, penis) contributed less than 0.6% combined (Table 1). The cumulative-share structure shows the first two sites alone exceeding 90% of expenditure.

The aggregate estimate was robust to deliberate variation of the attribution assumption. Across the conservative, optimistic, and alternative-literature head-and-neck scenarios the total moved within ±6% of the base case, and even excluding the three head-and-neck sites entirely the aggregate remained EUR 44,558,433 (−17.7%). The cervical share rose, rather than fell, under every more conservative attribution, from 81.8% in the base case to 86.9% under the alternative head-and-neck values and 99.4% with head-and-neck sites excluded, so the compositional dominance of cervical cancer is not an artefact of the head-and-neck fractions about which transportability concern is greatest. The full scenario table is given in Supplementary S5.

### 3.2. Temporal Trend

HPV-attributable expenditure rose steeply and monotonically over the study period, from EUR 3,671,605 in 2021 to EUR 19,894,426 in 2025, a 5.4-fold increase corresponding to a 2021-indexed value of 542 (Table 2). Year-on-year growth was modest in 2022 (9.9%), accelerated abruptly in 2023 (140.0%), and then decelerated to 73.9% in 2024 and 18.0% in 2025. The descriptive generalised least squares smoothing of the monthly log-series gave an average increment of about 3% per month, an annualised factor near 1.46, reported without a confidence interval because the near-random-walk behaviour of the series leaves few independent observations (φ = 0.94; Section 2.9 and Supplementary S9).

**Figure 1:**
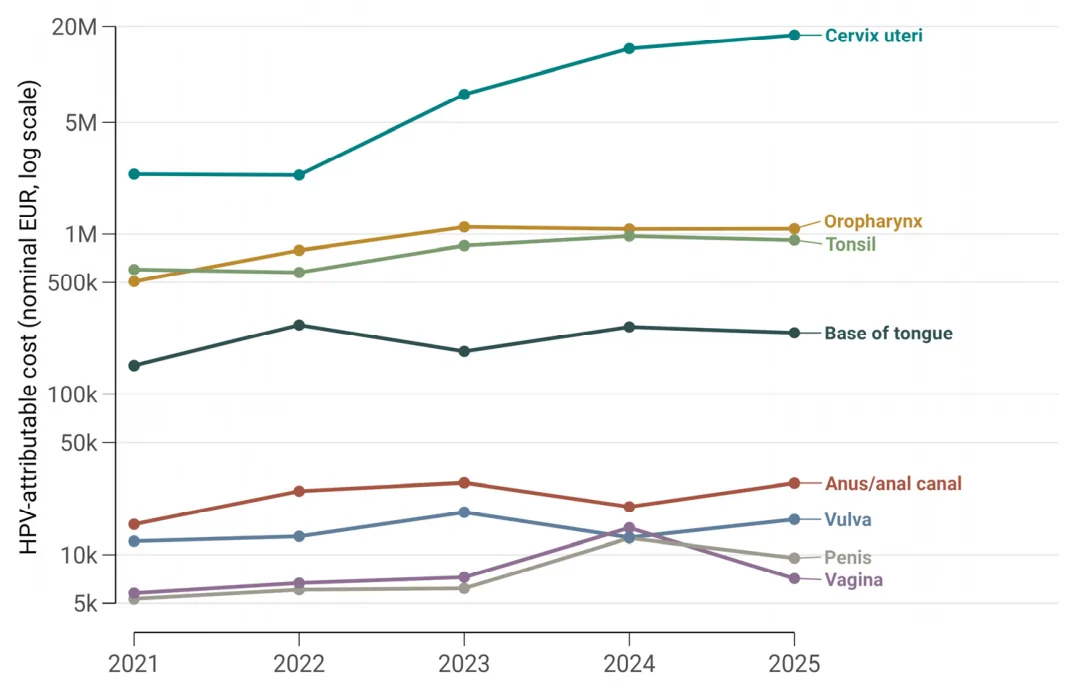
Annual HPV-attributable pharmaceutical expenditure by cancer site, NHIF Application 2, Bulgaria, 2021–2025, in nominal euros. Total expenditure rises from EUR 3.7 million to EUR 19.9 million. The cervical share rises from 64.7% to 88.4% while the head-and-neck sites contract in relative terms.

The proportional composition shifted concurrently, cervical cancer rising from 64.7% of annual HPV-attributable expenditure in 2021 to 88.4% in 2025 while the head-and-neck sites contracted in relative terms (Figure 1).

The growth was not an artefact of general price inflation or of population change. Re-expressing the all-site series in constant 2021 euros using the Bulgarian all-item HICP (cumulative inflation 30.4% over 2021–2025) reduced the increase from 5.4-fold nominal to 4.2-fold in real terms, leaving the trajectory steep and monotonic. In parallel, a denominator-based national series of expenditure per 100,000 represented population rose from EUR 54,630 in 2021 to EUR 315,068 in 2025 (an index of 577), tracking the nominal series closely because the represented population was near-constant, so that changes in represented population are insufficient to explain the observed increase. Both adjusted series are tabulated in Supplementary S4.

**Figure 2:**
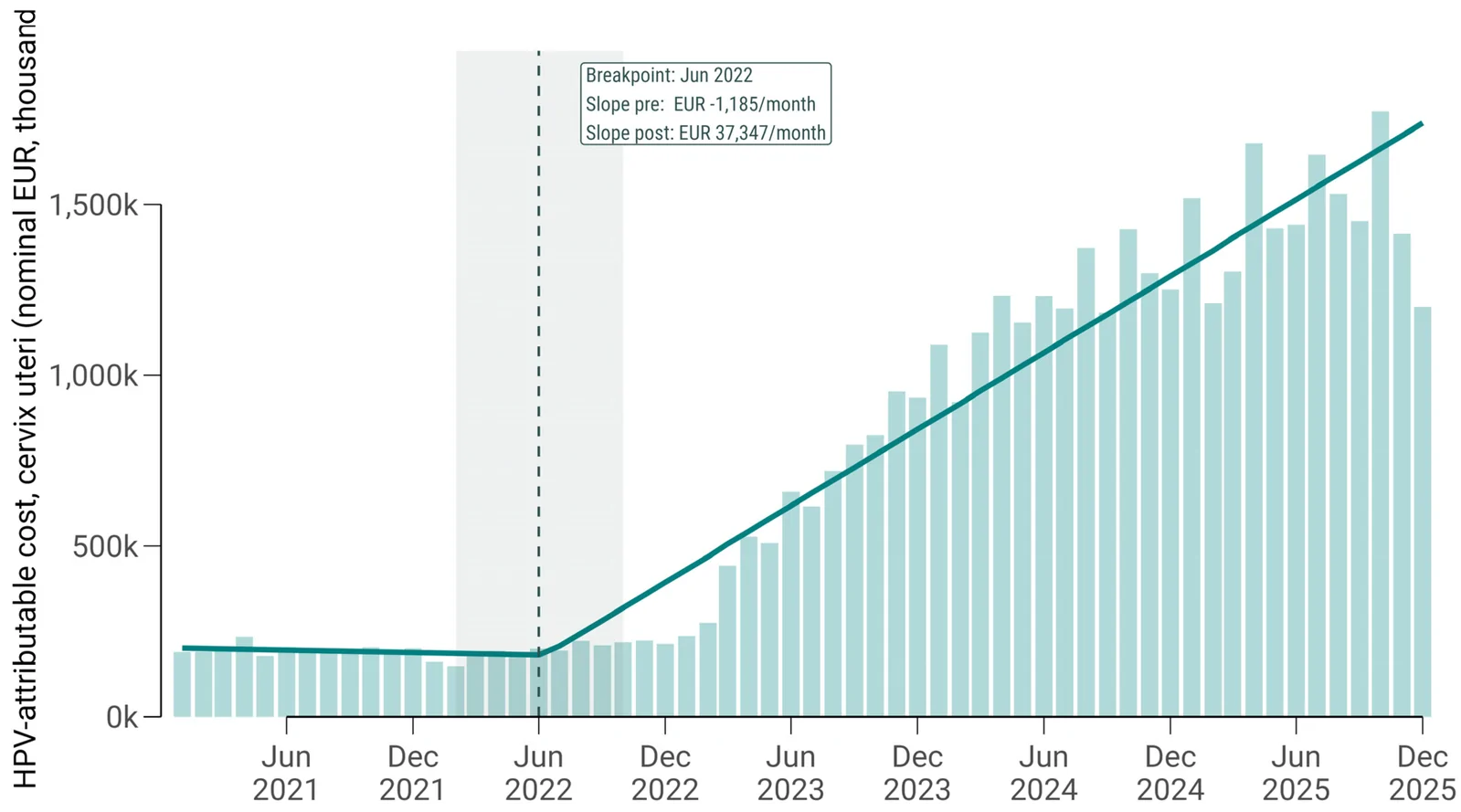
Monthly cervical cancer pharmaceutical expenditure (nominal euros) with an exploratory segmented-regression fit. The shaded band is the conditional 95% interval for the breakpoint location (February–October 2022) and is anticonservative. The breakpoint is a descriptive localisation rather than a confirmatory test. Pembrolizumab was first billed for cervical cancer in January 2023, within the post-breakpoint period of steep increase.

As a secondary and exploratory check on the timing of the cervical acceleration, segmented regression of monthly cervical expenditure located a single change in slope in mid-2022, near June 2022 on the natural scale and February 2022 on the log scale. This breakpoint precedes the first cervical billing of pembrolizumab in January 2023 by roughly six to eleven months, and the gap is expected rather than anomalous. A single-breakpoint fit locates the onset of the sustained upward slope, which began in 2022 as the reimbursed volume of established regimens recovered from the pandemic trough and biologic use widened, whereas pembrolizumab entered later and went on to dominate the magnitude of the rise rather than to initiate its slope. The two events are distinct and need not coincide. The estimated join is in any case imprecise, the two scales being reported side by side because the location is not tightly identified, and the conditional 95% interval (February–October 2022) is anticonservative because it assumes independent errors. The substantive content of the analysis is not the calendar placement but that phase-to-phase increases were far larger for the cervix (5.0-fold) than for any other site (1.4- to 1.9-fold), which indicates a cervical-specific rather than a system-wide shift (Figure 2).

### 3.3. Decomposition of Expenditure Growth

The price–volume–entry decomposition answers a single practical question: did expenditure rise because existing medicines became more expensive, because more of the same medicines were used, or because new and expensive medicines entered reimbursement (Table 3). Over the full 2021–2025 window, cervical expenditure grew 7.41-fold. This was concentrated overwhelmingly in net product entry (entry factor 5.93, equal to 88.8% of log growth), with the within-product volume effect contributing positively (Fisher quantity index 1.81) and the within-product price effect contributing negatively (Fisher price index 0.690, a roughly 31% decline in the reimbursement prices of products present in both years). The Törnqvist cross-check and the factor-reversal validation are reported in Supplementary S6. Because falling within-product prices masked part of the underlying expansion, the constant-price (real) cervical series rose even faster than the nominal series in volume-and-entry terms, reaching an index of 1075 by 2025 at 2021 prices against a nominal index of 741.

**Figure 3:**
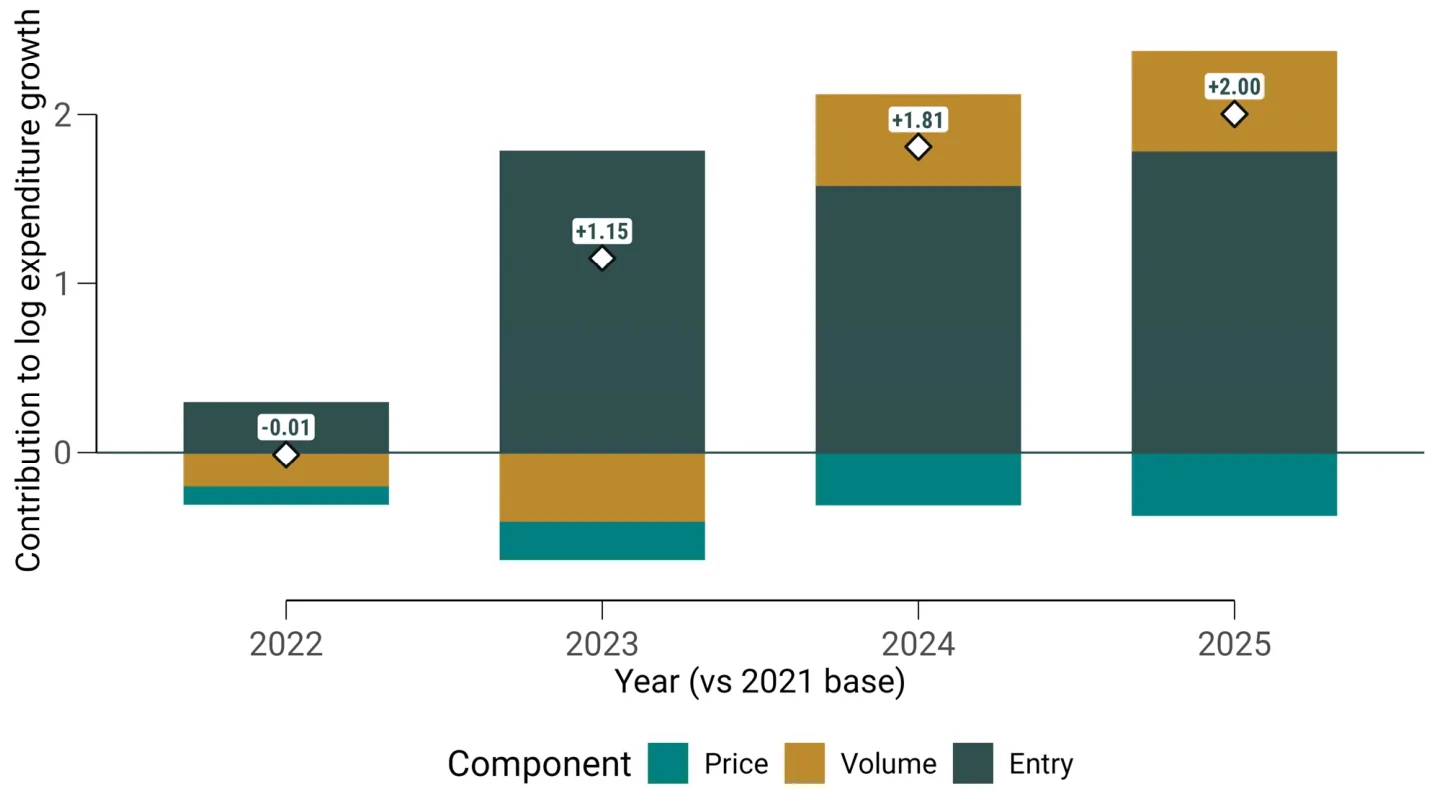
Decomposition of cervical cancer pharmaceutical expenditure growth into within-product price, within-product volume, and net product-entry components (cumulative log contributions versus the 2021 base, with diamonds denoting total log growth). Net product entry is the dominant positive component (88.8% of log growth by 2025), within-product volume contributes positively, and within-product price contributes negatively (reimbursement prices of established products fell by roughly 31%).

The product contributing most strongly to the entry effect was pembrolizumab. This agent (NHIF product code lh399, ATC L01FF02), absent from cervical reimbursement in 2021, first appeared in the cervical data in January 2023 and thereafter rose steadily, exceeding EUR 1.6 million in single months by late 2025. It accounted for EUR 16,028,911 of cervical expenditure in 2025, approximately 91% of cervical expenditure that year and more than four fifths of all HPV-attributable expenditure across every site, and EUR 33,509,830 cumulatively over the period. Its first billing thus post-dates the start of the series by two years, locating the entrant within the window of accelerating expenditure rather than at baseline. The remaining cervical entrants were predominantly bevacizumab presentations and supportive agents of far smaller magnitude, the full entrant and exit product tables, including the bevacizumab ATC reclassification, being given in Supplementary S7. By contrast, the per-site decomposition for the non-cervical sites showed value ratios between 1.24 and 2.13, driven by within-product volume growth with entry factors at or below unity, indicating that the head-and-neck and anogenital increases reflected wider reimbursed use of established regimens rather than new high-cost entrants (Figure 3).

## 4. Discussion

### 4.1. Principal Findings

Across five complete calendar years of national reimbursement data, the estimated inpatient pharmaceutical reimbursement attributable to HPV-related cancers in Bulgaria amounted to EUR 54.2 million and rose 5.4-fold in nominal terms, and 4.2-fold after adjustment for general consumer-price inflation, from EUR 3.7 million in 2021 to EUR 19.9 million in 2025. The expenditure was concentrated in cervical cancer (81.8% of the total) and, most strikingly, in a single molecule. The factor-reversal-validated decomposition showed that the growth was concentrated overwhelmingly in net product entry rather than in price inflation in established therapies, whose within-product reimbursement prices in fact fell by roughly 31%, or in rising reimbursed volumes of established regimens. The slope change in mid-2022 and the EUR 33.5 million cumulative cervical expenditure traced to pembrolizumab, first billed in January 2023 and reaching EUR 16.0 million in 2025 alone, together identify the reimbursement entry of checkpoint-inhibitor immunotherapy as the dominant accounting component of the cervical-specific acceleration. This is an attribution of expenditure to its accounting source, not a causal claim about why total spending rose. The decomposition establishes that growth occurred through the entry of a new product, and the chronology is consistent with that product being pembrolizumab, but the design does not isolate the contribution of reimbursement-policy changes, indication broadening, or procurement decisions that may have co-determined the timing. One further boundary governs the whole trajectory. The quantity measured is reimbursement expenditure under a single financing stream, which is distinct from total direct medical cost, from societal burden, and from epidemiological disease burden, so a rising series is not evidence of rising incidence. An unchanged or even declining case load could generate the same path once a costly molecule enters, and no inference about incidence, prevalence, or stage distribution is warranted.

### 4.2. Comparison with Prior Evidence

These findings extend the existing Bulgarian evidence base both qualitatively and quantitatively. Prior national work estimated the total economic burden of cervical cancer, including productivity losses on the order of EUR 7.6 million, and separately addressed the non-cervical HPV-associated sites [7,20], but it did not isolate the pharmaceutical component using complete reimbursement data, nor decompose its growth. The scale of the present estimate places that earlier work in sharper relief. Our inpatient antineoplastic estimate for cervical cancer alone (EUR 44.3 million over five years, reaching EUR 17.6 million in 2025) substantially exceeds the previously reported indirect-cost figure. It also exceeds the regional estimate of around EUR 7 million in future productivity loss attributed to Bulgarian HPV-related cancer deaths in the Central and Eastern European modelling of Sabale and colleagues [15], illustrating how rapidly the inpatient-drug component has come to dominate the cost profile of this disease.

Placed against the wider European cost-of-illness literature, the Bulgarian inpatient-drug total occupies an intermediate position once the difference in scope is taken into account. National studies that capture the full direct medical cost of HPV-related cancers report annual totals of roughly EUR 38–40 million in Norway [10] and, inclusive of productivity loss, approximately EUR 94 million in Sweden [11], while the broader Italian accounting of nine HPV-related conditions reaches EUR 529–543 million per year [13,17]. Our figure is restricted to the inpatient antineoplastic component under a single financing instrument and necessarily lies below such full-perspective totals, yet its rapid trajectory is consistent with the central message of that literature, namely the dominance of the cervical site and the rising contribution of biologic and immunotherapeutic agents [10,11,12,14]. The same compositional shift is visible beyond Europe in the most recent United States accounting, where higher per-case treatment cost has moved spending towards the treatment-dominated composition contrast noted in the Introduction [18]. What the present analysis contributes that national-aggregate descriptive studies cannot is a mechanism-level decomposition of expenditure growth, showing that for one middle-income payer the rise was carried almost entirely by the entry of a single new molecule rather than by price or volume movement in established therapy.

A second point of contrast concerns the unit of measurement. Most prior HPV cost-of-illness studies report per-patient or per-case costs from incidence-based or cohort designs [9,10,32], whereas the present design is prevalence-based and reports reimbursement administrations rather than persons. The two cannot be aligned directly, and the per-administration figures here should not be compared with per-patient costs elsewhere. The strength of the present design lies instead in completeness and in the internal identification of price, so that growth can be decomposed without recourse to external cost assumptions.

### 4.3. Immunotherapy Entry and Its Budgetary Implications

The pembrolizumab signal warrants attention because it sets clinical benefit against budgetary scale. Reimbursement for persistent, recurrent, or metastatic cervical cancer followed pivotal trial evidence of improved survival [33] and represents a real advance for a patient group with historically poor outcomes, the earlier entry of bevacizumab having established biologic-augmented therapy in this indication [34]. Modelled cost-effectiveness analyses at prevailing prices have nonetheless questioned the value of checkpoint-inhibitor regimens in this setting, with reported incremental cost-effectiveness ratios above conventional willingness-to-pay thresholds in several configurations [35,36]. Our data show that a single molecule came to account for the great majority of national cervical antineoplastic expenditure within three years of first reimbursement. The acceleration should not, however, be read as either uniquely Bulgarian or as the effect of one molecule operating independently of the wider oncology transition. The chronology is consistent with several non-exclusive mechanisms that the design cannot separate: the timing of the national reimbursement decision and any conditions attached to it, broadening of the reimbursed indication, adoption of the agent into treatment guidelines, and the gradual diffusion that characteristically follows market entry rather than a step change at the approval date. Each of these would produce the observed pattern of a delayed and then rising expenditure share, and the same first-generation checkpoint-inhibitor transition is unfolding across European oncology systems. What is specific to Bulgaria is the concentration of the effect in a single financing stream and a single disease site, not the underlying therapeutic shift. Although vaccination-related reductions in head-and-neck cancers lag cervical effects by decades, continued expansion of immunotherapy indications suggests this expenditure pressure may persist rather than plateau. This is a forward implication rather than a forecast, as the data do not extend beyond 2025.

### 4.4. Policy Implications

The policy implications follow, with appropriate caution about what the design can support.

First, the concentration of growth in one high-cost agent strengthens the case for value-based procurement and managed-entry agreements for oncology immunotherapies, instruments that permit access while containing budgetary exposure. Confidential price–volume and outcome-based agreements already operate within the managed-entry frameworks of several Central and Eastern European systems, including Hungary and Romania, and pooled procurement has been explored regionally to improve the negotiating position of smaller markets against single-source biologics. For a payer in which one molecule can dominate a disease-specific expenditure line within three years, the budgetary predictability of capped or risk-shared contracts is of direct fiscal value.

Second, the magnitude of the downstream expenditure documented here is consistent with, though it does not by itself establish, an economic rationale for the upstream prevention that Bulgaria has under-deployed, namely organised population-based cervical screening and substantially higher, ideally gender-neutral, HPV vaccination coverage in line with European and global elimination goals [4,6,37,38]. International cost-effectiveness evidence consistently finds routine adolescent vaccination cost-saving or highly cost-effective, while catch-up vaccination at older ages is far less favourable [39,40], which sharpens the case for restoring coverage in the cohorts where it remains effective. We did not model vaccine cost-effectiveness, counterfactual prevention savings, or avoided expenditure, and claim no quantified prevention return. The cost-of-illness parameter estimated here is intended as an input to exactly such future evaluation, and its full probabilistic draw distribution has been preserved so that a later cost-effectiveness model of gender-neutral HPV vaccination can resample the aggregate directly, propagating attributable-fraction uncertainty into the incremental cost-effectiveness estimate.

### 4.5. Strengths and Limitations

Several limitations are structural. First, the perspective is partial. NHIF Application 2 captures only antineoplastic and supportive pharmaceuticals dispensed outside the bundled clinical-pathway tariff, and it excludes pathway-bundled hospitalisation, radiotherapy, surgery, diagnostics, outpatient follow-up, primary care, and the substantial indirect costs of HPV-related cancers [15,16]. Total direct medical cost is therefore systematically underestimated, the excluded screening, diagnostic, and non-antineoplastic components being of a magnitude comparable to or larger than the antineoplastic component captured, as the United States accounting cited above indicates [18]. The component captured is nonetheless the inpatient antineoplastic one widely regarded as the fastest-growing element of oncology expenditure, so the partial perspective isolates the driver of greatest policy concern. Second, the data carry no unique patient identifiers, staging, or clinical outcomes. Counts are reimbursement administrations rather than persons, and high-cost agents for advanced disease inflate per-administration cost in later years independently of patient volume, which is why growth was disaggregated by product entry rather than read from aggregate trends. Being administrative records, the data are also shaped by coding practice, procurement and pricing arrangements, and treatment-eligibility criteria and their evolution over time, none of which can be reconstructed from the released extract and any of which can move recorded expenditure independently of the care delivered. Third, the attribution is ecological, applying internationally pooled and locally unvalidated fractions to aggregate expenditure without tumour-level HPV ascertainment. The probabilistic analysis addresses parameter uncertainty in the fractions only, not the structural and measurement uncertainty (miscoding, reimbursement heterogeneity, and geographic and temporal variation in HPV positivity at the head-and-neck sites), so the probabilistic interval is narrower than the true uncertainty of a national attribution. The discrete scenarios mitigate this concern, the aggregate moving within ±6% across the conservative, optimistic, and alternative-literature scenarios and remaining above EUR 44 million with the head-and-neck sites removed while cervical dominance was preserved or strengthened, but the non-cervical estimates remain subject to non-quantified transportability error. Fourth, early-pandemic suppression of treatment initiation may have depressed 2020–2021 expenditure and exaggerated apparent growth, although 2020 was excluded entirely and the decomposition attributes growth to identifiable product entry rather than to a low baseline. Finally, the monthly trend is descriptive rather than inferential. It is fitted against few effectively independent observations, the log-series approximating a random walk (φ = 0.94) with residual autocorrelation that the first-order correction does not fully clear, so no confidence interval is placed on the monthly increment and the independent-error comparators are read only as an upper bound. The segmented-regression breakpoint is likewise an anticonservative location estimate, and the temporal argument accordingly rests on the annual series and the decomposition.

Against these limitations stand notable strengths. The analysis rests on complete national reimbursement data rather than a sample, applies a transparent and fully reproducible pipeline with hard integrity checks and unit-tested decomposition invariants, and, uncommonly in oncology cost-of-illness work, separates the price, volume, and entry components of growth using indices that satisfy factor reversal. This resolution allows expenditure growth to be attributed to product entry rather than to inflation or within-product reimbursement changes, an attribution of direct relevance to forecasting and procurement.

## 5. Conclusions

The estimated inpatient pharmaceutical reimbursement attributable to HPV-related cancers in Bulgaria grew rapidly between 2021 and 2025, in real as well as nominal terms, and was concentrated in cervical cancer and in a single immunotherapeutic agent, with the increase occurring through product entry rather than through price or volume changes in established therapies. The trajectory reflects the reimbursement of costly therapies rather than any measured change in disease frequency, in a setting where prevention coverage remains incomplete. In an era of expanding immunotherapy indications, these findings provide a quantified, mechanism-resolved expenditure parameter for budget-impact forecasting, for procurement and managed-entry policy, and for the economic evaluations of HPV-related cancer prevention that the data here are intended to inform.

## Figures and Tables

**Table 1:** HPV-attributable inpatient pharmaceutical expenditure by cancer site, full period 2021–2025 (nominal euros). Administration columns count reimbursement administrations per facility–product–diagnosis–month stratum, not unique individuals. Low and high bounds reflect one-way PAF sensitivity (cervical PAF fixed at 1.00).

Cancer Site	Strata (n)	Administrations (HPV-adj.)	Cost HPV-adj. (EUR)	95% PAF Interval (EUR)	% of Total	Cumulative %
Cervix uteri	26,812	46,763	44,288,937	44,288,937	81.8	81.8
Oropharynx	3522	3305	4,564,964	3,912,827–5,217,102	8.4	90.2
Tonsil	3438	3054	3,915,471	3,413,488–4,417,455	7.2	97.4
Base of tongue	1208	899	1,111,514	952,727–1,270,302	2.1	99.5
Anus/anal canal	1711	1641	115,605	109,390–121,821	0.2	99.7
Vulva	1567	1138	72,621	62,247–82,995	0.1	99.8
Vagina	758	615	41,484	36,165–46,802	0.1	99.9
Penis	1163	564	39,786	31,321–48,251	0.1	100.0
**Total**	**40,179**	**57,981**	**54,150,383**	**52,807,101–55,493,666**	**100.0**	—

**Table 2:** Annual HPV-attributable expenditure, all sites combined (nominal euros), with the year-on-year change and the nominal and HICP-deflated real indices (2021 = 100).

Year	Cost HPV-adj. (EUR)	95% PAF Interval (EUR)	Year-on-Year Change (%)	Index (2021 = 100) *
2021	3,671,605	3,496,168–3,847,043	—	100
2022	4,034,839	3,803,641–4,266,037	+9.9	110
2023	9,694,378	9,394,383–9,994,373	+140.0	264
2024	16,855,134	16,531,656–17,178,613	+73.9	459
2025	19,894,426	19,581,254–20,207,599	+18.0	542

* Cumulative consumer-price inflation over the period was 30.4%, so the real series rises 4.2-fold against the 5.4-fold nominal series and general inflation accounts only for the gap between the two. The full real-terms and per capita series are given in Supplementary S4.

**Table 3:** Fisher price–volume–entry decomposition of cervical cancer expenditure, fixed 2021 base (multiplicative factors, with the entry log-contribution share).

Comparison *	Value Ratio	Price (Fisher)	Volume (Fisher)	Entry Factor	Price Contrib. (%)	Volume Contrib. (%)	Entry Contrib. (%)
2021→2023	3.16	0.794	0.666	5.97	−20.1	−35.3	155.0
2021→2024	6.10	0.733	1.72	4.85	−17.2	29.8	87.3
2021→2025	7.41	0.690	1.81	5.93	−18.6	29.7	88.8

* The 2021→2022 comparison is omitted here because total growth that year was essentially nil (value ratio 0.99), which makes the percentage decomposition numerically unstable and uninformative. That comparison is retained with an explanatory note, alongside the full per-year price, volume, and entry contribution shares, in Supplementary S6.

## Data Availability

The NHIF inpatient pharmaceutical reimbursement dataset is publicly available via Zenodo (DOI: 10.5281/zenodo.19160637). Population denominators are available from the National Statistical Institute open-data portal. The analysis code is deposited in the associated public repository.

## Supplementary Materials

The following supporting information can be downloaded at: https://www.mdpi.com/article/10.3390/healthcare14162474/s1, The following are provided as Supplementary File S1: the data-preparation and attrition table (S1); the population-attributable fractions and site scope (S2); the probabilistic sensitivity analysis summary, with the full 10,000-draw vector persisted for cost-effectiveness hand-off (S3); the HICP real-terms and national per capita expenditure series (S4); the discrete PAF attribution scenarios (S5); the full price–volume–entry decomposition with factor-reversal validation, including the cervical fixed-base and per-site results (S6); the cervical entrant and exit product tables and the monthly pembrolizumab trajectory, with an explicit note on the bevacizumab ATC reclassification (S7); the ATC canonicalisation audit (S8); the trend-model specification and diagnostics, including the autoregressive parameter (S9); the completed CHEERS 2022 and RECORD checklists (S10, S11); and a reproducibility statement describing the pipeline integrity checks and pinned environment (S12). The full analysis code and pinned environment lockfile are deposited on GitHub (Data Availability). Supplementary File S2: session record.

## Abbreviations

The following abbreviations are used in this manuscript:

AR (1)	First-order autoregressive
ATC	Anatomical Therapeutic Chemical (classification)
CC BY	Creative Commons Attribution
CHEERS	Consolidated Health Economic Evaluation Reporting Standards
CI	Confidence interval
CIN	Cervical intraepithelial neoplasia
CEE	Central and Eastern Europe
DOI	Digital object identifier
EUR	Euro
GEO	Geographical (Eurostat dimension code)
GLS	Generalised least squares
HICP	Harmonised Index of Consumer Prices
HPV	Human papillomavirus
HTA	Health technology assessment
ICD-10	International Classification of Diseases, 10th Revision
ICER	Incremental cost-effectiveness ratio
NA	Not applicable
NHIF	National Health Insurance Fund
NSI	National Statistical Institute
OLS	Ordinary least squares
PAF	Population-attributable fraction
PSA	Probabilistic sensitivity analysis
QALY	Quality-adjusted life-year
STROBE	Strengthening the Reporting of Observational Studies in Epidemiology
WTP	Willingness to pay
YLL	Years of life lost
