# Supplementary material for: Estimated HPV-Attributable Inpatient Pharmaceutical Reimbursement in Bulgaria, 2021–2025: A National Payer-Perspective Expenditure Analysis
Source: Healthcare (Basel). 2026 Aug 10;14(16):2474. doi: 10.3390/healthcare14162474 (PMC13512374; doi:10.3390/healthcare14162474)
Supplement: Supplementary file 1 [file healthcare-14-02474-s001.zip › healthcare-4391011-File S1.pdf]

Supplementary File S1

Supplementary Materials

Kostadin Kostadinov [1,2]*, Victoria Mandova [1], Vanya Rangelova [3], Ani Kevorkyan [3] and Ralitsa Raycheva [1]

[1] Department of Social Medicine and Public Health, Faculty of Public Health, Medical University of Plovdiv, 4002 Plovdiv, Bulgaria
[2] Environmental Health Division, Research Institute at Medical University of Plovdiv, 15-A "Vasil Aprilov" Blvd., 4002 Plovdiv, Bulgaria
[3] Department of Epidemiology and Disaster Medicine, Faculty of Public Health, Medical University of Plovdiv, 4002 Plovdiv, Bulgaria
* Correspondence: kostadinr.kostadinov@mu-plovdiv.bg

This document provides the supplementary tables, the extended methodological detail, and the reporting checklists referenced in the main text. To avoid duplication, the rationale for each analytic choice is given in the main-text Methods. The material here is the numerical detail and validation output that the main text points to rather than a restatement of method. All monetary values are in euros (EUR). Patient counts are reported as billing administrations per facility–product–diagnosis–month stratum and are not unique-person counts (Main text, Limitations). Unless otherwise stated, every figure reproduced here is taken directly from the analysis log generated by the single reproducible pipeline, with the random seed for the probabilistic analysis fixed at 2026.

S1. Data Preparation and Attrition

The source extract is the National Health Insurance Fund (NHIF) inpatient pharmaceutical reimbursement database (Application 2, Извън стойността на КП, "outside the value of the clinical pathway"), publicly deposited on Zenodo (DOI: 10.5281/zenodo.19160637) [1]. The documented unit of observation is the facility–product–diagnosis–month stratum defined by regional health insurance office, contracted hospital, NHIF product code, ICD-10 diagnosis, and calendar month. A stratum may carry more than one billing line, and the release does not deduplicate these. The full preparation sequence is reported in Table S1, expanding the description given in the main-text Methods (Data preparation). The step-by-step count table is provided here rather than in the main text.

The analysis was restricted to the five complete calendar years 2021–2025 (60 months). The July–December 2020 records present in the Zenodo release were excluded at source so that every year contributes twelve months and no partial-year artefact enters the temporal analysis. Within the restricted window, rows were loaded and the period field validated as a date with no missing values. Zero-cost records were removed. Multi-line strata were then collapsed to the documented stratum key by summing the additive payment attributes, a collapse verified to be loss-free for total reimbursed expenditure (delta below EUR 10-6) and to render the stratum key unique. Cost outliers were reviewed but not excluded. Records exceeding ten times the 99th percentile of the reimbursed amount (threshold EUR 477,284, against a 99th percentile of EUR 47,728) were flagged for inspection. Ten records met this criterion, all were clinically plausible high-cost biologic administrations, none fell within the HPV-attributable ICD-10 scope, and all were therefore retained without effect on the analysis.

Several data-handling questions bear on the completeness of the extract and are documented here in full. The product-code, quantity (pack_num), and reimbursed-amount fields contained no missing values across the 943,169 lines, and the reimbursed amounts contained no negative entries, corrections, or reversals, so the exclusion at step 1 removed lines with a cost of exactly zero rather than any credit or adjustment record. A missing ICD-10 diagnosis was present in 6,698 lines. Because the HPV filter matches a site-specific

regular expression, a missing code cannot enter the HPV scope, and these lines remain only in the all-diagnoses total that supports the denominator (the 1.86% share of Application 2 expenditure). Of the 588 zero-cost lines removed at step 1, 24 fell within the HPV-attributable ICD-10 scope, and six of those carried a non-zero pack quantity. Those six lines contributed six packs in total, 0.0040% of the 150,137 packs in the HPV-scope expenditure-positive data, so their removal is immaterial to the volume decomposition. Retaining a zero-priced but non-zero-quantity line would have introduced a per-pack price of zero into the Fisher price index, so the exclusion additionally protects the internal price identification. The 2,267 multi-line strata identified at step 3, each carrying at most two lines, were summed to the documented stratum key rather than deleted, a reconciliation to the publisher's analytical unit that is loss-free for total expenditure.

An outlier review analogous to the global one was conducted within the HPV scope, addressing the largest strata that drive the cervical entry result. No HPV-scope stratum reached the global flag threshold of EUR 477,284. The largest was a single-month pembrolizumab cervical administration of EUR 178,528 (62 packs, per-pack reimbursement EUR 2,879). Across all HPV-scope pembrolizumab lines the per-pack reimbursement was tightly clustered (interquartile range approximately EUR 2,867 to EUR 2,893, full range EUR 2,804 to EUR 3,068), consistent with the single reimbursed unit price of the product and providing no evidence of data-entry outliers in the strata responsible for the entry effect.

Table S1. Data preparation and attrition. Percentages are of the relevant prior total. The HPV-scope dataset of 40,179 strata is the basis for all attributable-cost estimates in the main text

Step	Operation	Records/Strata	Note
0	Rows loaded (2021–2025 window)	943,169	`period` confirmed date, no NAs
1	Exclude zero-cost records (`costs_eur` = 0)	−588 (0.06%)	942,581 remaining
2	Cost-outlier review (10 × p99 = EUR 477,284)	10 flagged, 0 excluded	all outside HPV scope, retained
3	Collapse multi-line billing lines to stratum key	942,581 → 940,314	2267 multi-line strata merged, loss-free for cost
—	Cleaned analytic dataset (`df_nhif_clean`)	940,314 strata	period 2021-01 to 2025-12
4	Filter to HPV-attributable ICD-10 scope	40,179 (4.273%)	eight eligible sites (S2)

ICD-10 codes were upper-cased and whitespace-trimmed before filtering with the regular expression `^C53|^C51|^C52|^C21|^C60|^C01|^C09|^C10`. Because the NHIF Application 2 dataset covers invasive malignancies receiving antineoplastic treatment, carcinoma-in-situ codes do not appear and require no explicit exclusion.

S2. Population-Attributable Fractions and Site Scope

The eight eligible cancer sites and their base-case and bounding population-attributable fractions (PAFs) are reproduced in Table S2. The main text gives the compact version of this table (Table S1) and the attribution rationale; the values here carry the full bounding columns used downstream. The fractions are drawn from pooled international literature [2–6]. The cervical fraction is fixed at unity because persistent oncogenic HPV infection is the necessary cause of cervical carcinoma, and it is not varied in any analysis. Larynx (C32) and oral cavity (C02–C06) are excluded from the base case because their HPV-attributable fraction is substantially lower and more heterogeneous, and the consequence of this exclusion is addressed by the head-and-neck attribution scenarios in S5 rather than by a separate inclusion analysis.

Table S2. Site-specific population-attributable fractions. The base case applies the point value, while the low and high columns give the one-way deterministic bounds carried into the probabilistic sensitivity analysis (S3).

Cancer Site	ICD-10 Prefix	PAF Base	PAF Low	PAF High
Cervix uteri	C53	1.00	1.00	1.00
Vulva	C51	0.70	0.60	0.80
Vagina	C52	0.78	0.68	0.88
Anus/anal canal	C21	0.93	0.88	0.98
Penis	C60	0.47	0.37	0.57
Base of tongue	C01	0.70	0.60	0.80
Tonsil	C09	0.78	0.68	0.88
Oropharynx	C10	0.70	0.60	0.80

S3. Probabilistic Sensitivity Analysis: Aggregate Draw Distribution

Parameter uncertainty in the PAFs was propagated through a Monte Carlo simulation of 10,000 draws (seed 2026). For each non-cervical site the PAF was sampled from a Beta distribution moment-matched to its point estimate, with the deterministic low and high bounds (S2) treated as an approximate 95% interval, while the cervical PAF was held at unity in every draw. Each draw produced an aggregate HPV-attributable cost as the sum across sites of observed cost multiplied by the sampled fraction. The resulting distribution is summarised in Table S3.

Table S3. Aggregate HPV-attributable expenditure: deterministic base case and probabilistic distribution (10,000 draws, seed 2026). The interval is narrow because cervical cancer, whose fraction is fixed at unity, dominates the aggregate. It is therefore driven entirely by the non-cervical sites and should not be read as capturing structural or measurement uncertainty (Main text, Limitations).

Quantity	Value (EUR)
Deterministic base case	54,150,383
Probabilistic median	54,161,894
Probabilistic 2.5th percentile	53,292,429
Probabilistic 97.5th percentile	54,950,063

The full 10,000-element draw vector has been persisted (`output/paf_psa_draws.rds`) so that a subsequent cost-effectiveness model of gender-neutral HPV vaccination can resample the aggregate HPV-attributable cost directly, propagating attribution uncertainty into the incremental cost-effectiveness estimate rather than treating the burden as a fixed point input.

S4. HICP Real-Terms and National Per-Capita Series

Two adjustments distinguish genuine growth in reimbursement from artefacts of general price inflation and of population change. The main text reports the nominal and real indices in compact form within the annual table (Table S4) and states the conceptual distinction between the HICP deflator and the internal Fisher price index. This section provides the full underlying series.

S4.1 Real-Terms (HICP-Deflated) Series

The nominal series was re-expressed in constant 2021 euros using the Bulgarian all-items Harmonised Index of Consumer Prices (HICP; Eurostat series prc_hicp_aind, classification CP00, GEO = Bulgaria) [7]. The annual-average index was rebased to 2021 = 100

directly from the published index values (data vintage 6 February 2026), giving cumulative consumer-price inflation of 30.4% over 2021–2025 (Table S4).

Table S4. All-sites HPV-attributable expenditure in nominal and constant 2021 euros, deflated by the Bulgarian all-items HICP. The nominal 5.4-fold increase corresponds to a 4.2-fold real increase, so general inflation accounts only for the gap between the two indices.

Year	Nominal (EUR)	HICP Index (2021 = 100)	Real, Constant 2021 EUR	Nominal Index	Real Index
2021	3,671,605	100.0	3,671,605	100	100
2022	4,034,839	113.0	3,570,654	110	97
2023	9,694,378	122.7	7,899,720	264	215
2024	16,855,134	125.9	13,386,794	459	365
2025	19,894,426	130.4	15,251,622	542	415

S4.2 National Per-Capita Series

A denominator-based national series expressed annual HPV-attributable expenditure per 100,000 of the represented resident population, using National Statistical Institute mid-year estimates aggregated over the 16 NHIF regional offices that bill under a code in the released extract. The Pernik office bills under no code and its population is excluded so that numerator and denominator span the same offices. Because the regional field encodes the administering office of the treating hospital rather than patient residence, this series is a service-utilisation density and is not an incidence rate (Table S5).

Table S5. National HPV-attributable expenditure per 100,000 represented resident population. The per-capita index (577) tracks the nominal index closely because the represented population was near-constant, indicating that population change is insufficient to explain the rise.

Year	Expenditure (EUR)	Represented population	EUR per 100,000	Index (2021 = 100)
2021	3,671,605	6,720,914	54,630	100
2022	4,034,839	6,335,964	63,682	117
2023	9,694,378	6,334,449	153,042	280
2024	16,855,134	6,327,474	266,380	488
2025	19,894,426	6,314,336	315,068	577

S5. Discrete PAF Attribution Scenarios

Because the probabilistic analysis (S3) samples around fixed published values and therefore addresses parameter rather than structural uncertainty, four discrete scenarios vary the attribution assumption itself (Table S6). The main text summarises the result that the aggregate moves within ±6% across scenarios and that cervical dominance is preserved or strengthened. This section gives the full scenario table and the values substituted. The conservative and optimistic scenarios apply the published lower and upper PAF bounds to all non-cervical sites. The alternative-literature scenario substitutes lower head-and-neck fractions (oropharynx 0.45, base of tongue 0.45, tonsil 0.55) to reflect documented geographic heterogeneity in HPV positivity at these sites. These substituted values are a deliberately conservative reading of the head-and-neck literature and should be confirmed against the cited source before final submission. The head-and-neck-excluded scenario sets the fractions for C01, C09, and C10 to zero, isolating the cervical and anogenital burden least subject to transportability concern. The cervical PAF is fixed at unity throughout.

Table S6. Aggregate HPV-attributable expenditure under discrete attribution scenarios, full period. The aggregate moves within ±6% across the conservative, optimistic, and alternative-literature scenarios and remains above EUR 44 million with head-and-neck sites removed, while cervical dominance is preserved or strengthened in every scenario.

Scenario	Aggregate (EUR)	vs. Base (%)	Cervical Share (%)
Base (published point PAFs)	54,150,383	—	81.8
Conservative (non-cervical PAF low bounds)	52,807,101	−2.5	83.9
Optimistic (non-cervical PAF high bounds)	55,493,666	+2.5	79.8
Alternative-literature head-and-neck PAFs	50,968,507	−5.9	86.9
Head-and-neck sites excluded (PAF = 0)	44,558,433	−17.7	99.4

S6. Price–Volume–Entry Decomposition: Full Results and Validation

S6.1 Method and Validation

The decomposition method, including the definition of volume as reimbursed pack quantity and the factor-reversal property of the Fisher indices, is described in the main-text Methods. This section reports the validation output and the full per-year and per-site results that the main text summarises. The decomposition was verified against synthetic panels with known invariants, and the factor-reversal identity (the product of the Fisher price and quantity indices over the matched sample equals the matched value ratio, and the entry factor carries the matched ratio to the total value ratio) was confirmed numerically for every comparison, with the maximum logarithmic gap below 10^{-6}. ATC canonicalisation reduced the product dictionary to one ATC code and name per NHIF product code, resolving 190 distinct codes after harmonising the current WHO ATC reclassification (S8).

S6.2 Cervical Decomposition, Fixed 2021 Base

Table S7 reports the full fixed-base decomposition of cervical expenditure, including the per-year price, volume, and entry contribution shares omitted from the compact main-text table (Table S4). Over 2021–2025 cervical expenditure grew 7.41-fold, concentrated in net product entry (entry factor 5.93), with a positive within-product volume effect (1.81) and a negative within-product price effect (0.690, equivalent to a ~31% fall in the reimbursement prices of products present in both years).

Table S7. Cervical cancer price–volume–entry decomposition, fixed 2021 base. Note on the 2021→2022 row: this comparison is omitted from the compact main-text decomposition table and retained here for completeness. Total growth that year was essentially nil (value ratio 0.99), so the denominator of the percentage decomposition (total log growth) is near zero and the percentage contributions (price 837%, volume 1,579%, entry −2,315%) become numerically unstable and must not be interpreted literally. The interpretable quantities are the multiplicative Fisher price, Fisher volume, and entry factors, which remain well behaved.

Comparison	Value Ratio	Price (Fisher)	Volume (Fisher)	Entry Factor	Real Value Ratio	Price Contrib. (%)	Volume Contrib. (%)	Entry Contrib. (%)
2021→2022	0.99	0.899	0.818	1.34	1.10	837	1,579	−2,315
2021→2023	3.16	0.794	0.666	5.97	3.98	−20.1	−35.3	155
2021→2024	6.10	0.733	1.72	4.85	8.32	−17.2	29.8	87.3
2021→2025	7.41	0.690	1.81	5.93	10.7	−18.6	29.7	88.8

S6.3 Per-Site Decomposition, 2021→2025

Table S8 reports the full-period decomposition for each site. Cervical cancer is the only site with a large entry factor. At every other site the value ratio (1.24–2.13) is carried

by within-product volume growth with entry factors at or below unity, indicating wider reimbursed use of established regimens rather than new high-cost entrants.

Table S8. Per-site price–volume–entry decomposition, 2021→2025.

Cancer site	Value Ratio	Price (Fisher)	Volume (Fisher)	Entry Factor	Matched Products (n)
Cervix uteri	7.41	0.690	1.81	5.93	51
Oropharynx	2.13	0.943	2.26	1.00	35
Anus/anal canal	1.80	0.965	2.74	0.683	25
Penis	1.80	0.961	1.96	0.954	20
Base of tongue	1.61	0.949	1.70	0.995	19
Tonsil	1.52	0.940	1.62	1.000	26
Vulva	1.36	0.979	1.42	0.978	30
Vagina	1.24	1.14	1.21	0.895	12

S7. Cervical Entrant and Exit Products, and the Pembrolizumab Trajectory

S7.1 Entrant Products

Table S9 lists the products billed for cervical cancer in 2025 that were absent in 2021, ranked by 2025 reimbursed value. Pembrolizumab (NHIF code lh399, ATC L01FF02) dominates, accounting for EUR 16,028,911 of 2025 cervical expenditure. The remaining entrants are predominantly bevacizumab presentations and supportive agents of far smaller magnitude.

Table S9. Top ten cervical entrant products by 2025 reimbursed value (17 entrants in total). Each row is one NHIF product code (the decomposition unit). Seven of the eight bevacizumab presentations appear in the top ten, the eighth (lh671, EUR 676) ranking fourteenth. The italicised memo row is not a product but a molecule-level subtotal of all eight bevacizumab codes, given for orientation. It totals EUR 780,822, or 4.9% of the pembrolizumab entry, so no bevacizumab presentation, and indeed bevacizumab in aggregate, approaches the magnitude of the single pembrolizumab entrant.

NHIF Code	ATC	Product	2025 Value (EUR)	2025 Packs	Unit Price (EUR)
lh399	L01FF02	pembrolizumab	16,028,911	5,622	2,851
lh581	L01FG01	bevacizumab	304,505	1,692	180
lh538	L01FG01	bevacizumab	301,060	1,167	258
lh582	L01FG01	bevacizumab	88,864	1,930	46
lh539	L01FG01	bevacizumab	46,774	773	61
lh697	L03AA13	pegfilgrastim	20,888	97	215
lh706	L01FG01	bevacizumab	19,010	162	117
lh705	L01FG01	bevacizumab	14,732	476	31
lh476	L03AA13	pegfilgrastim	9,708	48	202
lh670	L01FG01	bevacizumab	5,201	32	164
— memo —	L01FG01	all bevacizumab (8 codes)	780,822	6,252	—

S7.2 Exit Products and the Bevacizumab Reclassification

A material methodological caveat attaches to the entry and exit accounting. The products recorded as leaving cervical reimbursement after 2021 include several bevacizumab presentations under ATC code L01XC07, while the entrants in Table S9 include bevacizumab presentations under the newer code L01FG01. This reflects the WHO ATC reclassification of bevacizumab from L01XC07 to L01FG01 rather than genuine therapeutic exit and re-entry of the molecule. Because distinct NHIF product codes are treated as

separate products in the decomposition, such reclassifications and any biosimilar presentations register in the entry and exit components rather than as a within-product price change. This does not affect the cervical entry result, which is overwhelmingly driven by pembrolizumab, a genuinely new molecule under a single stable code. No bevacizumab presentation approaches the magnitude of the pembrolizumab entry.

S7.3 Monthly Pembrolizumab Trajectory

This trajectory is aggregated at the molecule level, by ATC code (L01FF02), summing across all NHIF product codes for pembrolizumab, whereas the entrant ranking in Table S9 is aggregated at the product-code level. The two coincide exactly here, the trajectory's 2025 totals reproducing the entrant table's pembrolizumab row to the euro (EUR 16,028,911) and to the pack (5,622), because pembrolizumab is reimbursed under a single canonical code (lh399) in these data. The legacy L01XC18 label on the same code was resolved to L01FF02 during ATC canonicalisation (S8). Were a pembrolizumab biosimilar to enter under a second code, the molecule-level trajectory would capture it while the single product-code entrant row would not, so the two views are equivalent only so long as the molecule maps to one code.

Pembrolizumab first appears in the cervical data in January 2023 and rises steadily thereafter (Table S10). Its first billing post-dates the start of the series by two years, placing the entrant within the window of accelerating expenditure rather than at baseline. The cumulative total reaches EUR 33,509,830 by December 2025.

Table S10. Selected months from the cervical pembrolizumab reimbursement trajectory (full 36-month series January 2023 – December 2025 available in the analysis log). Monthly billing exceeds EUR 1.6 million in single months by late 2025.

Period	Monthly Value (EUR)	Packs	Cumulative (EUR)
2023-01	23,864	8	23,864
2023-06	451,448	152	1,421,706
2023-12	755,130	258	5,111,032
2024-06	1,036,035	360	10,679,769
2024-12	1,130,364	394	17,480,920
2025-06	1,311,683	458	25,289,054
2025-10	1,617,085	572	31,152,324
2025-12	1,074,112	380	33,509,830

S8. ATC Canonicalisation Audit

The released extract occasionally records more than one ATC code or name for a single NHIF product code, partly because of the current WHO ATC reclassification of several oncology biologics (for example, bevacizumab from L01XC07 to L01FG01, and the L01XC immunoglobulin series to L01FF and L01FG). Before any product-level analysis, each NHIF product code was reduced to a single canonical ATC code and name. After canonicalisation, a hard check confirmed that every NHIF product code mapped to exactly one ATC code and one ATC name, and the procedure resolved the product dictionary to 190 distinct ATC codes. This canonicalisation is a precondition for the price–volume–entry decomposition, which treats the NHIF product code as the homogeneous unit within which a per-pack price is defined.

S9. Trend-Model Specification and Diagnostics

The model specifications and their rationale are given in the main-text Methods (Section 2.9). This section reports the fitted parameters and the diagnostics that justify treating the monthly trend descriptively rather than inferentially. The trend was summarised by a generalised least squares (GLS) regression of log expenditure on a calendar-month index (1–60) with a first-order autoregressive error structure. Gamma (log link), quasi-Poisson (log link), log-linear ordinary least squares, and negative-binomial fits were retained as independent-error comparators.

For the all-sites series the GLS smoothing returned a monthly cost ratio of 1.03, an average multiplicative increment of about 3% per month (annualised factor near 1.46). The residual diagnostics show why this is reported as a descriptive summary and not as an inferential estimate. The estimated autoregressive parameter was high ($\varphi = 0.94$), so the monthly log-series is close to a random walk and the effective number of independent observations across the 60 months is small. The normalised residuals showed a lag-1 autocorrelation of −0.37 and a Ljung–Box statistic still indicating residual structure ($p < 0.001$), the expected footprint of fitting a near-unit-root series across a mid-period regime change rather than evidence of an alternative trend. The nominal model-based interval (1.01–1.05) is therefore anticonservative and is not carried to the main text. A higher-order autoregressive or ARMA specification was not pursued, because the residual structure originates in the trend and the regime change rather than in the autoregressive order and would not yield a valid interval on 60 points spanning a structural break. Consistent with the slope being estimated against little independent signal, all four independent-error comparators returned a slightly higher monthly cost ratio of approximately 1.04, read only as an upper bound on the increment. The monthly cost ratio is a multiplicative change in expenditure and is not an incidence rate ratio. The substantive temporal argument does not rest on this model. The price–volume–entry decomposition (S6) and the exploratory segmented-regression location (main text) carry it.

The change in slope of monthly cervical expenditure was located with segmented (piecewise linear) regression estimating a single breakpoint. This was specified a priori as a single breakpoint and is treated as an exploratory localisation rather than a confirmatory test, the breakpoint confidence interval being conditional on independent errors and therefore likely anticonservative. The raw-scale fit placed the change around June 2022 (conditional 95% interval February–October 2022), and a log-scale robustness fit placed it slightly earlier, in February 2022. The six-month difference between the two fits is itself a reason to treat the location as approximate.

S10. CHEERS 2022 Checklist

Completed Consolidated Health Economic Evaluation Reporting Standards (CHEERS 2022) checklist [8]. Item locations refer to the main text unless a supplementary section (S-) is given. Items specific to full economic evaluations with modelled comparators (for example, incremental cost-effectiveness outcomes, model assumptions, and analysis of effectiveness) are marked not applicable, because this study estimates a cost-of-illness input parameter and does not itself model a comparator intervention.

Table S11. CHEERS 2022 reporting checklist.

#	Item	Location/Status
1	Title identifies the study as an economic evaluation / analysis	Title
2	Abstract: structured summary	Abstract
3	Background and objectives	Introduction

4	Health economic analysis plan	Methods (Study design); analysis pre-specified in pipeline
5	Study population	Methods (Data source); S1
6	Setting and location	Bulgaria, national NHIF; Methods
7	Comparators	Not applicable (no comparator intervention; expenditure analysis)
8	Perspective	Public payer (NHIF); Methods (Study design)
9	Time horizon	2021–2025, five years; Methods
10	Discount rate	Not applicable (no future costs/effects discounted; nominal and HICP-real series reported, S4)
11	Selection of health outcomes	Not applicable (no health-outcome valuation)
12	Measurement of outcomes	Expenditure (EUR) and billing administrations; Methods (Outcome derivation)
13	Valuation of outcomes	Reimbursed cost as recorded; Methods
14	Measurement and valuation of resources and costs	Methods (Outcome derivation); S1
15	Currency, price date, conversion	EUR; nominal with 2021 price date; HICP real-terms series, S4
16	Rationale and description of model	Not applicable (no decision-analytic model); decomposition method in Methods and S6
17	Analytics and assumptions	Methods (PAF, decomposition, statistical analysis); S2, S6, S9
18	Characterising heterogeneity	Per-site decomposition (S6); per-capita series (S4)
19	Characterising distributional effects	Not applicable
20	Characterising uncertainty	Probabilistic sensitivity analysis (S3); discrete scenarios (S5); deterministic bounds (S2)
21	Approach to engagement with patients/stakeholders	Not applicable
22	Study parameters	Methods; S2 (PAFs); S4 (HICP)
23	Summary of main results	Results; Abstract
24	Effect of uncertainty	Results (scenario robustness); S3, S5
25	Effect of engagement with patients/stakeholders	Not applicable
26	Study findings, limitations, generalisability, current knowledge	Discussion
27	Source of funding	Funding statement (main text)
28	Conflicts of interest	Declaration (main text)

S11. RECORD Checklist

Completed REporting of studies Conducted using Observational Routinely-collected health Data (RECORD) checklist [9], the reporting extension of the Strengthening the Reporting of Observational Studies in Epidemiology (STROBE) statement [10] for studies that use routinely collected administrative data. The study is a retrospective, longitudinal, ecological payer-perspective reimbursement-expenditure analysis and is not cross-sectional. The checklist below retains the STROBE items (Table S12) and adds the RECORD-specific extension items (Table S13).

Table S12. STROBE items, retained within the RECORD checklist.

#	Item	Location/Status
1	Title and abstract: design indicated; balanced summary	Title; Abstract
2	Background / rationale	Introduction
3	Objectives	Introduction (final paragraph)
4	Study design	Methods (Study design): payer-perspective expenditure analysis
5	Setting, locations, periods	Bulgaria, NHIF, 2021–2025; Methods

6	Participants / eligibility	Strata meeting HPV-scope ICD-10 criteria; Methods, S1
7	Variables (outcomes, exposures)	Expenditure, administrations, PAF, product code; Methods, S2
8	Data sources / measurement	NHIF Application 2 (Zenodo); Methods, S1
9	Bias	Ecological attribution, non-unique counts, partial perspective; Discussion (Limitations)
10	Study size	940,314 strata; 40,179 HPV-scope; S1
11	Quantitative variables / groupings	ICD-10 site grouping; calendar year/month; Methods
12	Statistical methods	GLS AR(1), Fisher decomposition, PSA, scenarios; Methods, S3–S6, S9
13	Participants (numbers at each stage)	Attrition table, S1
14	Descriptive data	Results (aggregate burden); S2
15	Outcome data	Results; tables S3–S7
16	Main results	Results; Abstract
17	Other analyses (subgroups, sensitivity)	Per-site decomposition, scenarios, real-terms, per-capita; S4–S6
18	Key results	Discussion (Principal findings)
19	Limitations	Discussion (Strengths and limitations)
20	Interpretation	Discussion
21	Generalisability	Discussion
22	Funding	Funding statement (main text)

Table S13. RECORD-specific extension items for studies using routinely collected administrative data.

RECORD Item	Description	Location/Status
1.1	Type of data and name of the databases in title or abstract	Title and Abstract: NHIF Application 2 inpatient reimbursement data
1.2	Geographic region and timeframe in title or abstract	Title and Abstract: Bulgaria, 2021–2025
1.3	Database linkage stated in title or abstract	No person-level linkage; aggregate population denominators only (Methods)
6.1	Population-selection codes or algorithms listed in detail	ICD-10 site regular expression; Methods (Cancer site scope); S1, S2
6.2	Validation of the selection codes or algorithms referenced	No local code-validation study available; attribution fractions from pooled literature (S2); addressed as a limitation (Discussion, Strengths and limitations)
6.3	Flow diagram for the data-linkage process	Not applicable (no person-level linkage); attrition sequence in S1
7.1	Complete list of codes and algorithms for variables	ICD-10 site codes (S2), HPV regular expression (S1), ATC canonicalisation (S8)
12.1	Extent of investigator access to the database population	Complete public national extract; full access (Methods, Data source; S1)
12.2	Data-cleaning methods	Methods (Data preparation); S1 (zero-cost, outliers, ATC canonicalisation, collapse)
12.3	Linkage across databases and linkage-quality evaluation	Aggregate NSI-to-NHIF regional-office crosswalk; no person-level linkage; Methods (Population denominators)
13.1	Selection of records, including data-quality filtering	Attrition table (S1); Methods (Data preparation)
19.1	Implications of using data not created for research	Discussion (Strengths and limitations): coding practice, eligibility change, ecological misclassification
22.1	Access to supplemental information, raw data, and code	Data Availability: Zenodo dataset, public repository, pinned lockfile; S12

S12. Reproducibility

The complete analysis is implemented in a single reproducible R pipeline (hpv-nhif-analysis.R, Supplementary File S2) that runs end to end from the public Zenodo extract to every table and figure reproduced here. The pipeline applies the integrity checks described in the main-text Methods, including the loss-free verification of the stratum collapse, the factor-reversal numerical check on the decomposition, and the hard one-to-one ATC canonicalisation check. The random seed for the probabilistic analysis is fixed at 2026, and the environment is pinned through an accompanying lockfile.

References

1. Kostadinov, K. NHIF Inpatient Pharmaceutical Reimbursement Dataset, Bulgaria, 2020–2025 (Application 2, Izvan stoynostta na KP) 2026.
2. Martel, C. de; Plummer, M.; Vignat, J.; Franceschi, S. Worldwide Burden of Cancer Attributable to HPV by Site, Country and HPV Type. *International Journal of Cancer* **2017**, *141*, 664–670, doi:10.1002/ijc.30716.
3. International Agency for Research on Cancer *Biological Agents. IARC Monographs on the Evaluation of Carcinogenic Risks to Humans, Volume 100B*; International Agency for Research on Cancer: Lyon, France, 2012;
4. Abramowitz, L.; Lacau Saint Guily, J.; Moyal-Barracco, M.; Bergeron, C.; Borne, H.; Dahlab, A.; Bresse, X.; Uhart, M.; Préaud, E.; Asubin, S. Epidemiological and Economic Burden of Potentially HPV-Related Cancers in France. *PLoS ONE* **2018**, *13*, e0202564, doi:10.1371/journal.pone.0202564.
5. Hylin, H.; Thrane, H.; Pedersen, K.; Kristiansen, I.S.; Burger, E.A. The Healthcare Costs of Treating Human Papillomavirus-Related Cancers in Norway. *BMC Cancer* **2019**, *19*, 426, doi:10.1186/s12885-019-5596-2.
6. Östensson, E.; Fråberg, M.; Liljeström, B.; Helm, C.; et al. Cost of Preventing, Managing, and Treating Human Papillomavirus (HPV)-Related Diseases in Sweden. *PLoS ONE* **2017**, *12*, e0179520, doi:10.1371/journal.pone.0179520.
7. Eurostat Harmonised Index of Consumer Prices (HICP) – Annual Data (Average Index and Rate of Change), Bulgaria, All-Items (CP00) 2026.
8. Husereau, D.; Drummond, M.; Augustovski, F.; et al. Consolidated Health Economic Evaluation Reporting Standards 2022 (CHEERS 2022) Statement. *Value in Health* **2022**, *25*, 3–9, doi:10.1016/j.jval.2021.11.1351.
9. Benchimol, E.I.; Smeeth, L.; Guttmann, A.; Harron, K.; Moher, D.; Petersen, I.; Sørensen, H.T.; Elm, E. von; Langan, S.M. The REporting of Studies Conducted Using Observational Routinely-Collected Health Data (RECORD) Statement. *PLoS Medicine* **2015**, *12*, e1001885, doi:10.1371/journal.pmed.1001885.
10. Elm, E. von; Altman, D.G.; Egger, M.; et al. The Strengthening the Reporting of Observational Studies in Epidemiology (STROBE) Statement. *The Lancet* **2007**, *370*, 1453–1457, doi:10.1016/S0140-6736(07)61602-X.

Disclaimer/Publisher's Note: The statements, opinions and data contained in all publications are solely those of the individual author(s) and contributor(s) and not of MDPI and/or the editor(s). MDPI and/or the editor(s) disclaim responsibility for any injury to people or property resulting from any ideas, methods, instructions or products referred to in the content.
